# Rare and Elusive: The Challenges of Bronchoscopically Diagnosing Pulmonary Mucosa‐Associated Lymphoid Tissue Lymphoma

**DOI:** 10.1155/crpu/6756203

**Published:** 2026-03-08

**Authors:** Hamad Nasim, Robert Willim, Jason Beattie, Adnan Majid, Mihir Parikh, Kai Swenson

**Affiliations:** ^1^ Department of Surgery, Division of Thoracic Surgery and Interventional Pulmonology, Beth Israel Deaconess Medical Center, Harvard Medical School, Boston, Massachusetts, USA, harvard.edu; ^2^ Department of Pathology, Beth Israel Deaconess Medical Center, Harvard Medical School, Boston, Massachusetts, USA, harvard.edu

## Abstract

Primary pulmonary lymphomas (PPLs) are rare clinical entities representing 0.5%–1% of all pulmonary neoplasms, of which mucosa‐associated lymphoid tissue (MALT) lymphoma is the most common type (80%). PPLs are traditionally considered challenging to diagnose bronchoscopically given the rarity of these entities, small sample sizes, and the ancillary testing often required to adequately differentiate them from benign intraparenchymal lymph nodes. Here, we report three cases of pulmonary MALT lymphomas. In two cases, the patients presented with pulmonary nodules and were diagnosed by robotic‐assisted bronchoscopy following prior nondiagnostic biopsy attempts. The third patient presented with distal tracheal thickening, where flexible bronchoscopy revealed a particularly rare case of MALT lymphoma with endobronchial involvement.

## 1. Introduction

MALT lymphomas are low‐grade marginal zone B cell lymphomas and the most common type of PPL. They comprise 8% of the adult cases of non‐Hodgkin lymphoma, with the lungs as the second most common site (15%) of all MALT lymphomas after the gastrointestinal tract [1, 2].

Computed tomography (CT) findings in patients with pulmonary MALT lymphoma usually show bilateral consolidations or nodules with air bronchogram or ground glass attenuation. In cases where there is endobronchial involvement, imaging can show either a solitary intraluminal nodule, multiple nodular protrusions, or central airway thickening [3, 4].

In this case series, we expand on existing literature by highlighting the limitations of conventional bronchoscopy for diagnosing pulmonary MALT lymphoma and the role of advanced bronchoscopic techniques, such as robotic assisted bronchoscopy and transbronchial cryobiopsy, for obtaining adequate tissue samples for diagnosis. We also include a rare endobronchial presentation to emphasize the diverse ways in which the disease may present.

## 2. Case 1

A 76‐year‐old woman with chronic obstructive pulmonary disease and an active history of smoking was being followed up for a 1.6‐cm left upper lobe (LUL) lung nodule (Figure 1a) that was discovered on lung cancer screening. A CT‐guided transthoracic biopsy done at the time was nondiagnostic; however, on follow‐up, the nodule was found to be mildly hypermetabolic. She denied respiratory symptoms, fevers, or weight loss. She then underwent robotic‐assisted bronchoscopic sampling of the nodule. Transbronchial needle aspiration (TBNA) with a 21G needle and transbronchial biopsies using forceps were obtained. Linear endobronchial ultrasound (EBUS) examination revealed mediastinal adenopathy, which was biopsied using a 21G needle. There were no periprocedural complications. Pathological examination of the forceps biopsy demonstrated B cell predominant lymphoid aggregates (Figure 2a), and cytogenetic analysis revealed trisomies of Chromosomes 3 and 18, suggesting marginal zone lymphoma. TBNA from the lesion and lymph nodes was nondiagnostic, though flow cytometry and cytogenetics were not performed. Based on these reports, she was diagnosed with a low‐grade bronchus‐associated lymphoid tissue (BALT) lymphoma. She was referred to the primary hospital where she is being followed‐up; however, no further information was accessible.

Figure 1(a) CT scan showing a 1.6‐cm nodule in the LUL. (b) Multicystic lesion in the LLL.

**Figure:**
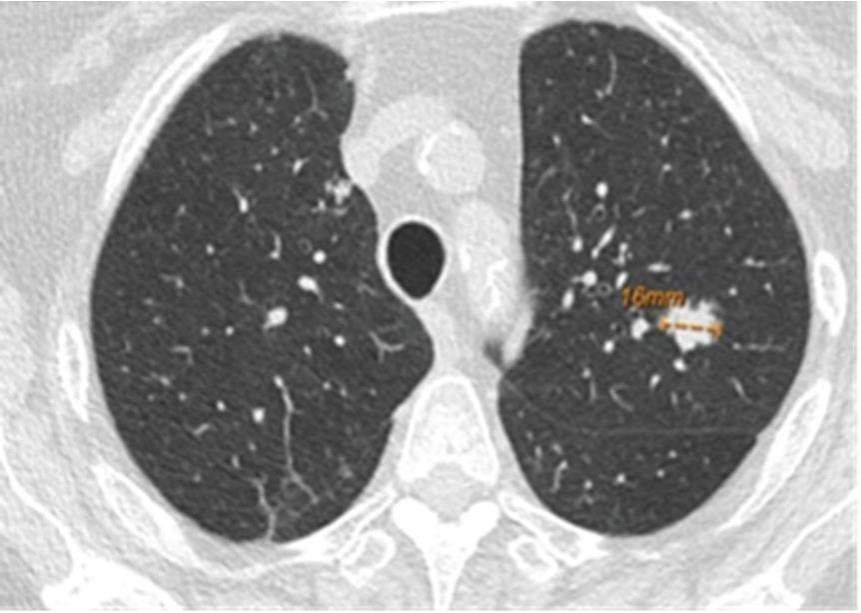
(a)

**Figure:**
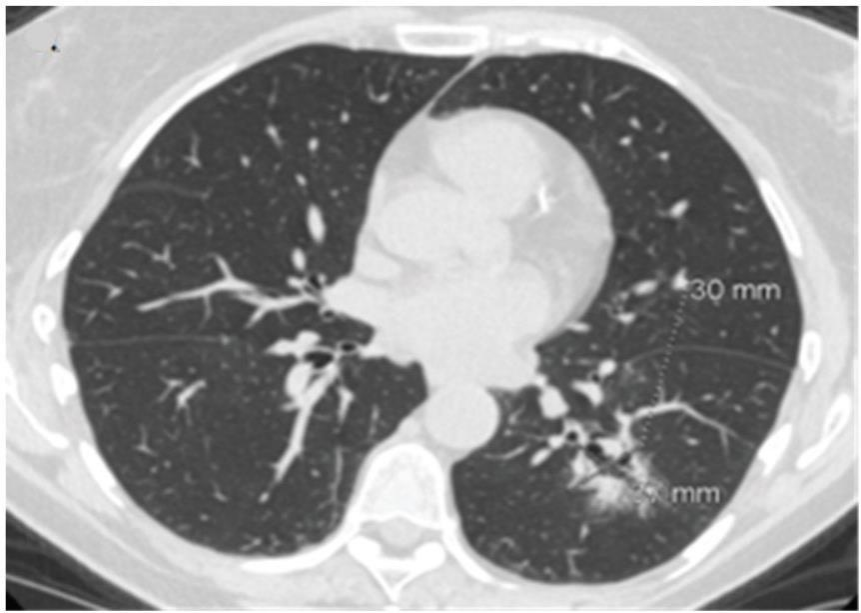
(b)

Figure 2(a) H&E section showing dense lymphoid infiltrates with medium‐ to small‐sized cells having irregular nuclear contours and a moderate amount of cleared out cytoplasm (magnification: 100×) (left). These were diffusely positive for CD20 indicating B cell predominance (right). (b) H&E section showing dense tissue infiltration with small‐sized abnormal lymphoid cells having similar characteristics as Figure 2a (magnification: 100×) (left). These were positive for PAX‐5 staining, indicating B cell predominance (right).

**Figure:**
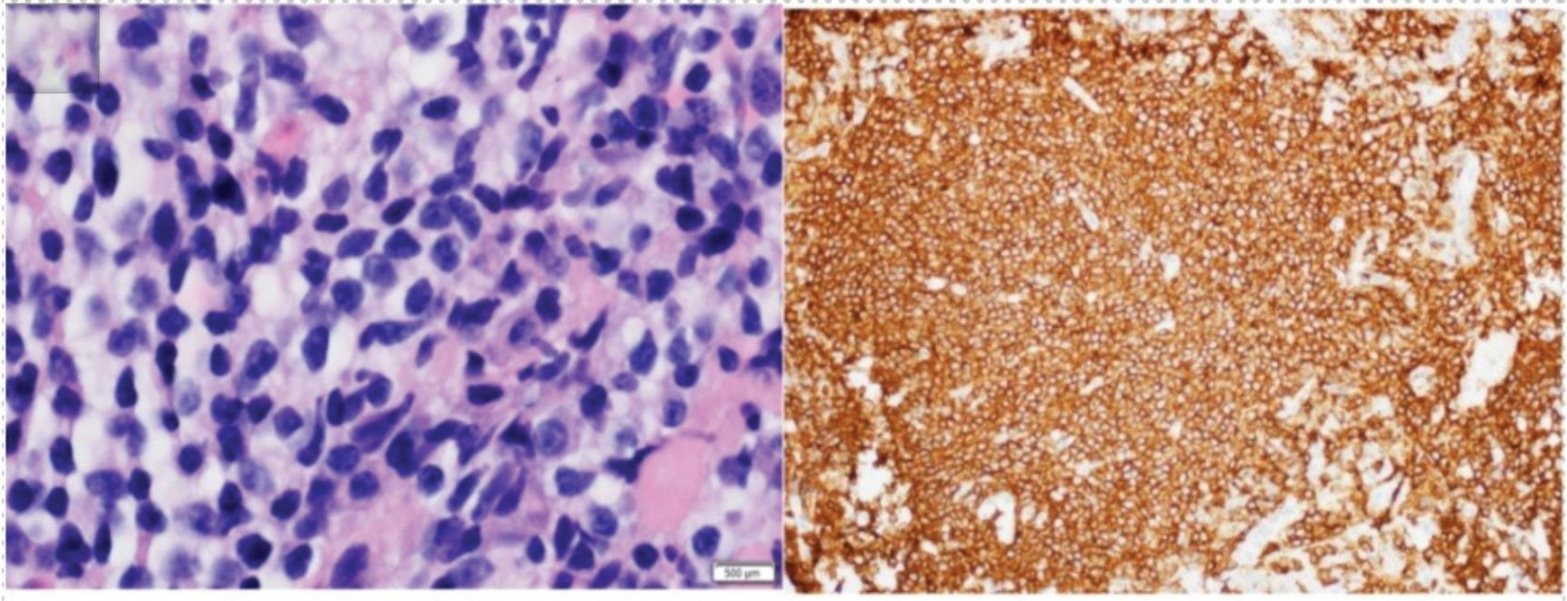
(a)

**Figure:**
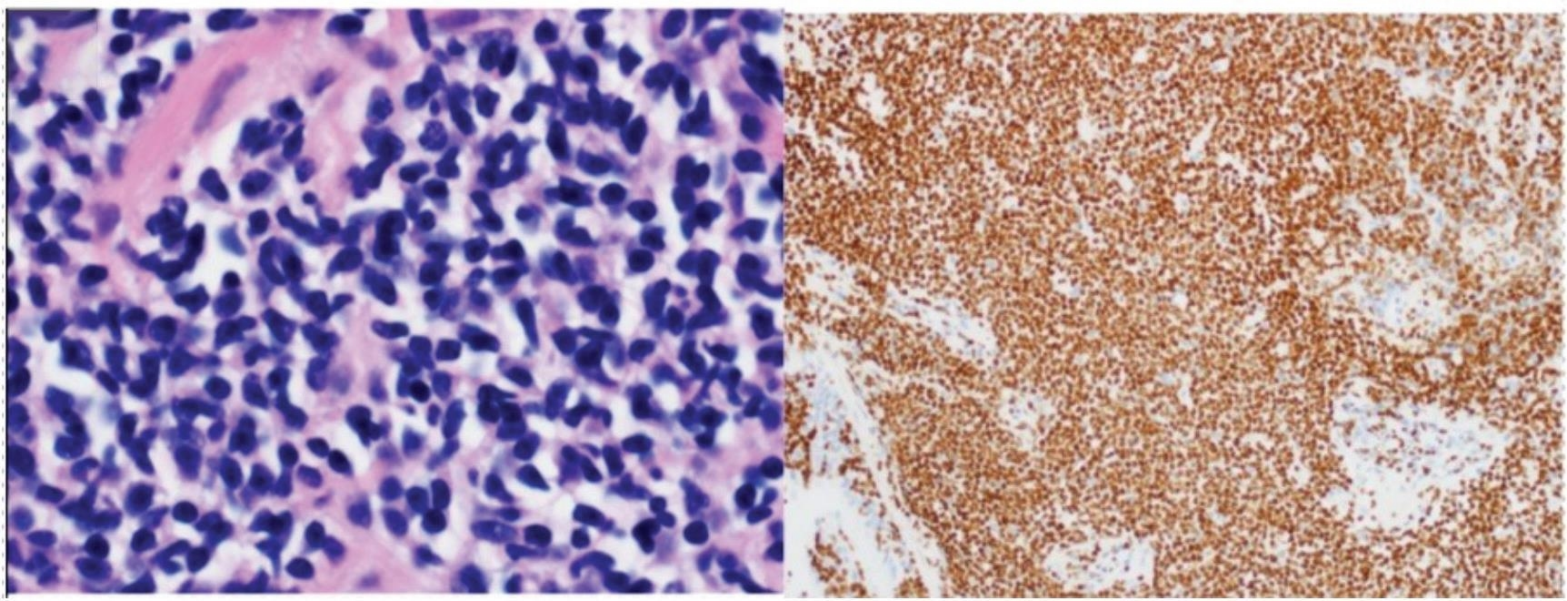
(b)

## 3. Case 2

A 66‐year‐old woman with a history of smoking was being evaluated for an incidentally discovered 2.0 × 1.6‐cm multicystic, spiculated left lower lobe (LLL) lung mass. She had undergone a computer‐guided bronchoscopic forceps biopsy and TBNA of the lesion 2 years ago, which was unremarkable. Follow‐up CT scan showed an increase in lesion size to 3.7 × 3.0 cm (Figure 1b) raising concerns for malignancy. She denied any respiratory or systemic complaints. She underwent a robotic‐assisted bronchoscopic sampling of the nodule, which included multiple TBNAs using a 21G needle and transbronchial cryobiopsies using a 1.1‐mm cryoprobe. Mediastinal adenopathy was revealed on linear EBUS and was biopsied using 21G needles. TBNA of the lesion revealed atypical lymphoid aggregates, and pathological examination of the cryobiopsies showed predominant B cell lymphoid aggregates (Figure 2b). Cytogenetic analysis showed BIRC3::MALT1 gene rearrangement. TBNA of the lymph nodes was unremarkable. Based on these reports, a diagnosis of low‐grade BALT lymphoma was made. Given the indolent nature of the disease and lack of symptoms, she is being followed‐up with radiological surveillance.

## 4. Case 3

A 65‐year‐old woman was being followed‐up for a 9‐mm LUL lung nodule and was found to have CT findings suggestive of distal tracheal and mainstem bronchi thickening. She is a nonsmoker with a history of left soft palate MALT lymphoma, left parotid mucoepidermoid carcinoma, right parotid marginal lymphoma, and Sjogren syndrome.

Flexible bronchoscopy revealed sessile lesions present in the right upper lobe (RUL), LUL as well as a mid‐tracheal outpouching (Figure 3a). Biopsies of these lesions were taken with flexible forceps, all of which showed dense lymphoid infiltrates (Figure 3b) of B cell origin indicating endobronchial MALT lymphoma. EBUS examination revealed no significant adenopathy. There were no periprocedural complications. Cytogenetic analysis of the RUL specimen was negative for a high‐grade lymphoma panel. Given the indolent nature of the disease, the patient was advised to continue follow‐up with her oncologist, where she is undergoing surveillance imaging with no therapy initiated at 2‐year follow‐up.

Figure 3(a) Bronchoscopy showing polypoid RUL endobronchial lesion. (b) Histology demonstrating dense lymphoid infiltrates.

**Figure:**
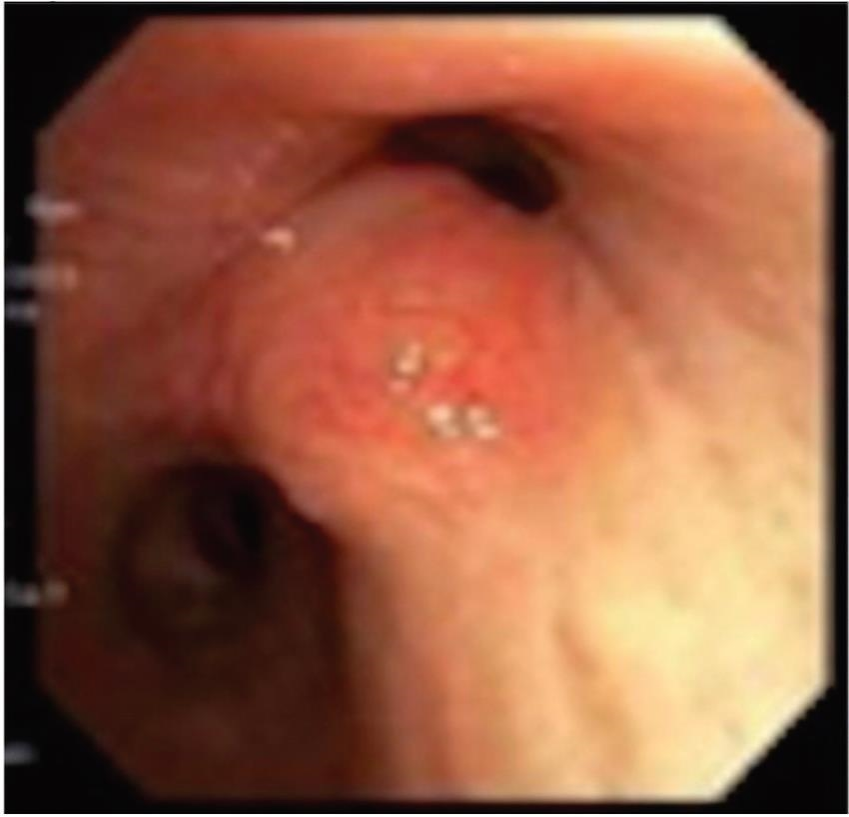
(a)

**Figure:**
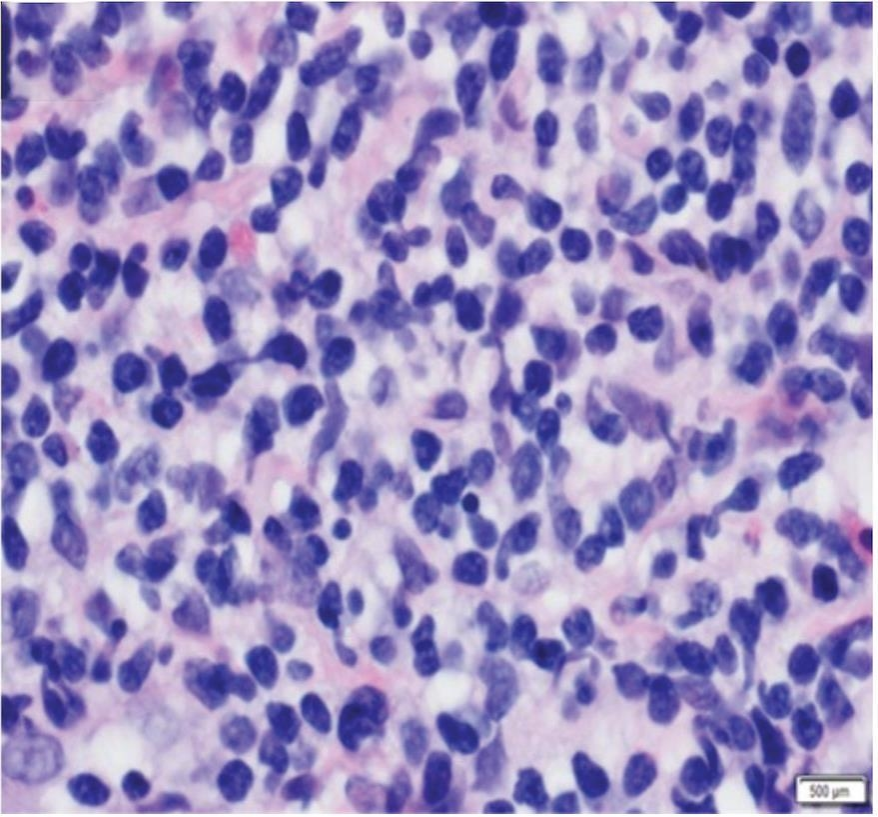
(b)

## 5. Discussion

The median age of presentation of pulmonary MALT lymphoma is 50–60 years with women being affected as often as men. It has an indolent clinical course and a good prognosis with a 10‐year survival rate of 72% and progression‐free survival of 36%. There are no clearly defined prognostic factors for MALT lymphoma; however, age (> 60 years) and performance status have been associated with shorter survival [2]. As in our cases, most patients are asymptomatic and symptoms, when present, are usually nonspecific such as cough, dyspnea, and hemoptysis. B symptoms are rare and only seen in aggressive forms of the disease [1, 5].

Even though pulmonary MALT lymphoma has no identifiable causative agent, it has been known to be associated with autoimmune diseases, such as Sjogren syndrome. A study showed up to 16% of patients had an autoimmune disease at the time of diagnosis [2].

Pulmonary MALT lymphoma is mostly a peripheral disease with endobronchial involvement being very rare (1.3%). In such cases, bronchoscopy findings include multiple wide‐stalked polyps similar to what was seen in Case 3. In contrast, other major primary endobronchial tumors usually show solitary polyps [6].

Due to the rarity of the condition, diagnosing and managing pulmonary MALT lymphoma is challenging. Tissue biopsy is the gold standard for diagnosis, and often additional specimens are required to be sent for histology as well as flow cytometry and cytogenetic/molecular analysis. We acknowledge that ancillary testing was incomplete in Case 1; however, a diagnosis of MALT lymphoma was made based on characteristic histopathologic features that showed dense infiltration of abnormal B lymphocytes and cytogenetic findings that indicated trisomies of Chromosomes 3 and 18.

Extrapulmonary involvement can be seen in up to 50% of the cases, most of which involve the gastric mucosa and bone marrow [1].

Bronchoscopic methods of obtaining tissue samples include EBUS‐guided TBNA and transbronchial biopsies. Literature indicates that while TBNA can be suitable for diagnosing pulmonary lymphomas overall, diagnosing certain subtypes including MALT lymphomas is difficult due to the small tissue sample size [7]. Transbronchial biopsies are usually utilized for diagnosing pulmonary MALT lymphomas. A retrospective study by Borie et al. showed that EBUS‐guided transbronchial forceps biopsy was able to provide a diagnosis in 23 out of the 26 patients [2]. Case reports have documented transbronchial biopsies obtained by cryoprobes, and they have been found to have better tissue quality as compared with forceps, with preserved architecture, fewer artifacts, and larger sample size [8, 9].Shape‐sensing robotic‐assisted bronchoscopy is increasingly being utilized to sample peripheral pulmonary lesions that may be difficult to access with conventional bronchoscopy. A multicenter study showed that bronchoscopists find this to be a useful platform, which helps localization and biopsy of peripheral tumors owing to its ability to navigate to and maintain a stable catheter at the target lesion, while allowing the passage of multiple sampling tools without deviating from the target [10]. This may be particularly helpful in cases of pulmonary MALT lymphoma where multiple adequate samples are often required for histopathologic evaluation and ancillary testing.

Treatment options for pulmonary MALT lymphoma include surgery and radiotherapy in localized disease, whereas in patients with more advanced disease, chemotherapy or immunotherapy can be used [1]; the relative efficacy of various treatment modalities is difficult to assess given the rarity of the condition and absence of comparative trials. In some cases, a conservative approach of surveillance imaging may be the best option in the setting of advanced age, comorbidities, limited disease, and lack of symptoms [11].

## 6. Conclusion

Our case series highlights the challenges of diagnosing pulmonary MALT lymphomas owing to its rarity, indolent nature, and often falsely negative biopsy results. We see here that in asymptomatic patients with persistent pulmonary nodules or central airway thickening, the possibility of MALT lymphoma should be considered.

## Funding

No funding was received for this manuscript.

## Disclosure

Abstract of these cases were presented as posters at AABIP 2024, Charlotte, NC, August 22–24. The first two cases were presented as a series in a single poster, and the third case was presented separately in another poster.

## Consent

No written consent has been obtained from the patients, as there is no patient identifiable data included in this case series.

## Conflicts of Interest

The authors declare no conflicts of interest.

## Data Availability Statement

The data that support the findings of this study are available on request from the corresponding author. The data are not publicly available due to privacy or ethical restrictions.
